# Summary of high field diffusion MRI and microscopy data demonstrate microstructural aberration in chronic mild stress rat brain

**DOI:** 10.1016/j.dib.2016.06.061

**Published:** 2016-07-05

**Authors:** Ahmad Raza Khan, Andrey Chuhutin, Ove Wiborg, Christopher D Kroenke, Jens R. Nyengaard, Brian Hansen, Sune Nørhøj Jespersen

**Affiliations:** a Center of Functionally Integrative Neuroscience, Aarhus University Hospital, Aarhus, Denmark; b Translational Neuropsychiatry Unit, Aarhus University, Risskov, Denmark; c Advanced Imaging Research Center, Oregon Health and Science University, Portland, OR, United States; d Stereology and Electron Microscopy Laboratory, Centre for Stochastic Geometry and Advanced Bioimaging, Aarhus University, Aarhus, Denmark; e Department of Physics and Astronomy, Aarhus University, Aarhus, Denmark

**Keywords:** Chronic mild stress, Diffusion MRI, Neurite, Histology

## Abstract

This data article describes a large, high resolution diffusion MRI data set from fixed rat brain acquired at high field strength. The rat brain samples consist of 21 adult rat brain hemispheres from animals exposed to chronic mild stress (anhedonic and resilient) and controls. Histology from amygdala of the same brain hemispheres is also included with three different stains: DiI and Hoechst stained microscopic images (confocal microscopy) and ALDH1L1 antibody based immunohistochemistry. These stains may be used to evaluate neurite density (DiI), nuclear density (Hoechst) and astrocytic density (ALDH1L1). This combination of high field diffusion data and high resolution images from microscopy enables comparison of microstructural parameters derived from diffusion MRI to histological microstructure. The data provided here is used in the article (Jespersen, 2016) [1].

**Specifications Table**

**Table:** 

Subject area	Neuroimaging
More specific subject area	Diffusion magnetic resonance imaging
Type of data	Excel files
How data was acquired	9.4T preclinical MRI system (Bruker, Germany). Confocal Microscope (Carl Zeiss, Germany) and Light Microscope DM6000B (Leica, Wetzlar, Germany)
Data format	Analyzed
Experimental factors	CMS rats divided into responders (anhedonic, *N*=6 ) and non-responders (resilient, *N*=8), plus age matched controls (*N*=7)
Experimental features	High field diffusion MRI data to explore microstructural alteration using biophysical modeling of diffusion MRI data and diffusion kurtosis imaging parameters. In addition, confocal microscopy was performed on the same hemisphere of each brain producing 3D stack images from amygdala. For this, the tissue was stained with DiI and Hoechst for neurite and nuclear density. Astrocyte density in amygdala was also evaluated using the ALDH1L1 antibody and light microscopy. Due to ineffective staining, astrocyte density data from two resilient animals are not provided and corresponding column left blank in the excel sheet
Data source location	Aarhus, Denmark
Data accessibility	Data are within this article as zip files. The full data set is available via Mendeley

**Value of the data**

• Data allows analysis of the relation between diffusion MRI based neurite density and histology based neurite density.
• Estimates of neurite, nuclear and astrocyte density from optical microscopy might provide better understanding or interpretation of diffusion MRI model parameters in terms of microstructural alteration.
• The data can be used for further analysis to explore microstructural alterations with other methods and in any brain regions.

## 1 Data

Data provided here has been used in the article [1].

The MR_parameters zip file contain excel files with the values of fit parameters of the biophysical model (neurite density (ν), extracellular diffusivity $(D_{\mathrm{eff}})$ and intra neurite diffusivity *(D*_L_)) [2], [3] as well as kurtosis fits (mean kurtosis (MK), mean diffusivity (MD), fractional anisotropy (FA), and the mean of kurtosis tensor (MKT)) [4], [5]. MR Zip files contain separate excel files with voxel values of each of the fitting parameters within ROIs (Prefrontal cortex (PFC), Hippocampus (HP), Caudate putamen (CP) and Amygdala (AM)) from rat brains belonging to groups of anh, res and ctr. Each individual excel file is named after the parameter it reports, and each sheet within the file is named <parameter>_<anh/res/ctr>_<ROI>. The histology zip file contains three excel files viz. astrocyte_density, neurite_density and nuclear_density, reporting histological data from amygdala. Each file has three excel sheets named ctr, anh and res. In both MR and histology data each column represents individual animal and row represents the value of MR parameter of single voxel in MR data, while row represents value of nuclear, neurite or astrocyte fraction of individual image in histology data.

The raw dataset is available through the following link and may be used by those interested in the implementation of alternative data analysis procedures:

https://data.mendeley.com/datasets/mh7sb75bsd/draft?a=453da998-dc8a-463e-9cbe-04f8e82a99a4.

## 2 Experimental design, materials and methods

Left hemispheres from a total of 21 brains of adult male wistar rats are contained in this data set. Of these, 7 brain hemispheres are from control rats and the remaining fourteen are from animals that underwent eight weeks exposure to the chronic mild stress paradigm described in [6]. These animals were then separated into eight resilient and six anhedonic on the basis of a sucrose consumption test. Rats were euthanized by exsanguination and the brains were fixed in paraformaldehyde solution until ex-vivo MRI data acquisition.

## 3 Diffusion MRI image acquisition and analysis

High field diffusion MRI data for biophysical modeling [2], [3] and for diffusion kurtosis imaging [4] was acquired on 9.4 T preclinical MR system (Bruker, Germany) using the combination of two protocols: 1) 14 nominal *b* values (*b*=0, 500, 1000, 1500, 2000, 2500, 3000, 3500, 4000, 4500, 5000, 6000, 7000, 8000 s/mm^2^) and 12 directions and 2) fast kurtosis dataset of nominal *b*=0, 1000, 2500 s/mm^2^and 9 specific directions as described in [5], [7], both with 250 µm isotropic voxels with TR=6500 ms, TE=26 ms, Δ/δ 15/5 ms, field of view (FOV) 25.5×12.5 mm and matrix size 102×50. High resolution T2 weighted images were acquired using a RARE (Rapid Acquisition with Relaxation Enhancement) sequence with 62.5 µm in plane resolution and 250 µm slice thickness. Identical slice positions were used for acquisition of *T*2 weighted and diffusion weighted images, so the former could be used for more precise ROI delineation without need of co-registration. The full data is provided raw (not normalized) along with gradient tables so that it may be analyzed using any fitting procedure (e.g. linear or non-linear least squares fitting procedures) [4], [5].

## 4 Confocal microscopy

Image stacks were obtained from amygdala with a confocal microscope equipped with a 63x/1.20 W Corr water immersion objective using an appropriate filter. The excitation wavelength for image acquisition from DiI and Hoechst stains was 549 and 480 nm respectively. Acquisitions of image stacks were performed using Zen2011 image processing software (Carl Zeiss).

## 5 Light microscopy

Immunohistological sections from amygdala were imaged with a Leica Microscope (Leica DM 6000). Whole tissue section montages were acquired with a 4X objective lens, and high resolution images were acquired with a 63x oil objective lens. Systematically sampled fields of views (FOV) of each section were taken within the amygdala region of the brain. Images were imported in ‘tif’ format for further image analysis in Matlab (The Mathworks, Natick, MA).
